# An intelligent SCADA-integrated deep learning framework for bird-safe offshore wind farm operation

**DOI:** 10.1038/s41598-026-55549-y

**Published:** 2026-06-13

**Authors:** Yara A. Sultan, Ahmed Sameh, Samah A. Gamel

**Affiliations:** 1 Mechatronics Department, Faculty of Engineering, Horus University, New Damietta, Egypt; 2 Production Engineering and Mechanical Design Department, Faculty of Engineering, Mansoura University, Mansoura, Egypt; 3 Electronics & Electrical Engineering Department, Faculty of Engineering, Horus University, New Damietta, Egypt

**Keywords:** Deep learning, Bird detection and classification, Offshore wind farms, SCADA systems, Avian collision mitigation, Environmental monitoring, Convolutional neural networks (CNNs), Renewable energy sustainability, Computer vision for wildlife protection, Intelligent turbine control, Ecology, Ecology, Energy science and technology, Engineering, Mathematics and computing

## Abstract

The rapid expansion of offshore wind energy has intensified concerns regarding avian collisions with turbine blades, particularly for migratory and high-risk bird species. Conventional mitigation approaches—including radar monitoring, manual intervention, and acoustic deterrents—are often limited by high false alarm rates, delayed response times, and the lack of species-level identification. To address these challenges, this study proposes an intelligent framework that integrates a Supervisory Control and Data Acquisition (SCADA) system with a Deep Convolutional Neural Network (DCNN)-based Bird Detection and Classification (BDC) model. The proposed system performs automated image-based bird classification and translates detection outputs into SCADA-driven turbine control actions through a multi-zone proximity assessment strategy. The model is trained and evaluated on a dataset comprising 525 avian species with over 90,000 images. Comparative analysis against conventional classifiers—including Support Vector Machines (SVM), Random Forest, K-Nearest Neighbor, and VGG16—demonstrates that the proposed BDC model achieves superior performance, with an accuracy of 99.62%, precision of 99.92%, recall of 100%, and an F1-score of 99.93%. In addition to classification performance, the system demonstrates a simulation-based system, achieving inference latency below 30 ms and SCADA response execution within 40 ms. These results highlight the potential of integrating deep learning with operational control systems to enable automated, risk-aware turbine response mechanisms for wildlife protection. It is important to note that the evaluation is conducted under controlled dataset conditions, and the dataset does not fully represent real offshore environments characterized by long-distance detection, motion blur, occlusion, and complex backgrounds. Therefore, the reported performance should be interpreted as an upper-bound estimate, and future validation using real-world offshore data is required to confirm deployment robustness. Overall, the proposed framework provides a simulation-based proof-of-concept approach for bridging AI-based avian monitoring with SCADA-enabled turbine control, contributing toward environmentally sustainable offshore wind farm operation.

## Introduction

The global transition toward low-carbon energy systems has accelerated the deployment of offshore wind farms, driven by the urgent need to reduce greenhouse gas emissions and decrease dependence on fossil fuels^1^. Wind energy has become one of the most technologically mature and economically competitive renewable resources, with significant growth in installed capacity over the last decade. Projections consistently indicate that offshore wind will constitute a major share of future global electricity generation owing to its favorable wind profiles, scalability, and reduced land-use constraints^2,3^. However, this rapid expansion has also amplified ecological concerns, particularly regarding its impact on avian species.

Bird collisions with wind turbines—primarily with the rotating blades—are well-documented sources of mortality for migratory birds, raptors, and seabirds^4^. In offshore environments, where large numbers of birds migrate seasonally, collision risks are intensified by increased rotor diameters, elevated hub heights, and reduced visibility conditions such as fog, low light, or offshore storms^5^. Previous assessments estimate that wind farms can cause hundreds of thousands of bird fatalities annually in some regions, although exact numbers vary due to monitoring limitations and environmental variability^6^. These concerns have prompted regulatory bodies, conservation agencies, and wind-energy operators to seek advanced mitigation solutions capable of balancing renewable-energy development with ecological protection.

Traditional bird monitoring and deterrence strategies—such as human visual surveys, acoustic repelling devices, periodic shutdowns, and radar-based monitoring—offer partial protection but suffer from limitations including restricted detection accuracy, weather sensitivity, operator dependency, and high operational costs^7–9^. Acoustic measures often fail in noisy marine environments, while radar systems may struggle to reliably classify species or differentiate birds from other aerial targets. More importantly, most existing systems operate reactively, initiating shutdowns only after a bird approach dangerously close, which increases collision risk and reduces the reliability of turbine-curtailment strategies.

Recent advances in artificial intelligence (AI) and computer vision have introduced new possibilities for automated wildlife monitoring. Deep learning–based classifiers, particularly convolutional neural networks (CNNs), have demonstrated superior capabilities in detecting and classifying birds from complex visual backgrounds, outperforming traditional machine-learning models such as Support Vector Machines (SVM), Random Forest (RF), and K-Nearest Neighbor (K-NN) in both accuracy and generalization^15–21^. Moreover, AI can process continuous video feeds and identify species in real time, enabling a shift from manual observation to fully automated, scalable monitoring frameworks. These developments are especially promising for offshore environments, where continuous human observation is infeasible.

Despite substantial progress in bird-classification research, relatively few studies integrate AI-based avian recognition with operational wind-farm control systems. The Supervisory Control and Data Acquisition (SCADA) system—widely used for monitoring turbine performance, environmental conditions, and operational safety—presents a unique opportunity for intelligent wildlife-protection strategies. SCADA’s real-time data acquisition and command-execution capabilities allow for automated turbine control, including speed reduction and emergency shutdowns, when external threats such as approaching birds are detected^12–14,23,25^. However, the integration of deep learning–based detection systems with SCADA operational logic remains underexplored and lacks comprehensive experimental evaluation.

To address this critical gap, this study presents an intelligent SCADA-integrated Deep Learning framework designed to detect, classify, and respond to bird presence around offshore wind turbines. The framework incorporates a Bird Detection and Classification (BDC) system based on a deep convolutional neural network (DCNN), coupled with a multi-zone proximity assessment model that triggers tiered responses—ranging from visual and acoustic warnings to automated turbine shutdowns—depending on the bird’s distance and flight trajectory. Using a high-quality dataset of 525 species comprising more than 90,000 images^29^, the proposed system is trained and benchmarked against widely used classifiers including SVM, Random Forest, VGG16, and K-NN. The results demonstrate that the proposed model significantly outperforms existing approaches, achieving a classification accuracy of 99.62% with strong precision, recall, and F1-scores.

The main contributions of this work are as follows:

1. *A unified end-to-end architecture* that integrates deep-learning-based bird classification with SCADA-level turbine control—representing, **simulation-based** proof-of-concept framework for intelligent avian-risk mitigation. .
2. *A multi-stage avian detection pipeline* comprising image acquisition, CNN-based species identification, proximity mapping, and adaptive risk-based turbine-response logic.
3. *A simulation-based response strategy strategy* that translates AI-driven detection outputs into turbine control actions, including automated derating and emergency shutdown when high-risk birds enter hazardous flight zones.
4. *A comprehensive comparative analysi* **s** of state-of-the-art bird-monitoring and classification approaches, highlighting the absence of operational integration in existing research^15–21^.
5. *A scalable system design with potential applicability to offshore environments*,* subject to future offshore validation* that leverages established SCADA communication protocols to ensure reliable deployment under harsh environmental conditions^12–14,23–27^.

By merging advances in deep learning with real-time SCADA control, this work introduces a new paradigm for environmentally conscious offshore wind-farm operation. The proposed framework enhances wildlife protection, supports regulatory compliance, and contributes to the sustainable expansion of offshore renewable energy.

## Related work

Research on bird–wind turbine interactions has expanded significantly with the global acceleration of offshore wind energy development. Existing literature can be broadly categorized into four main themes: (i) traditional monitoring and deterrence systems, (ii) radar- and sensor-based avian tracking, (iii) deep learning approaches for automated bird detection and classification, and (iv) integrated monitoring and control strategies for collision mitigation.

This section critically evaluates the state of the art across these domains and outlines the key limitations that motivate the SCADA-integrated framework proposed in this study.

### Traditional bird monitoring and deterrence approaches

Early mitigation strategies relied primarily on human-led visual surveys, mechanical deterrents, or environmental cues. Trained observers stationed onshore or aboard marine vessels monitored bird flight activity, assessed collision risks, and recommended temporary turbine shutdowns during peak migration periods^8^. Although effective in localized applications, manual monitoring is labor-intensive, subjective, and ill-suited for continuous offshore deployment due to harsh weather conditions, restricted visibility, and the high cost of field personnel.

Various deterrence mechanisms—such as acoustic repellents, pulsed lighting systems, and combined audiovisual alarms—have also been developed to divert birds from approaching turbine blades^9–11^. Pulsed lighting, commonly used at airports to prevent bird–aircraft collisions, has been adapted to wind farms, while acoustic systems employ high-frequency or ultrasonic signals. However, practical challenges persist: (i) reduced effectiveness in noisy offshore environments, (ii) the tendency of birds to habituate to repeated stimuli, and (iii) the inability of these systems to differentiate between high-risk and low-risk species.

A combined pulsed-light and acoustic system using white light and 2 kHz signals (90–135 dB) showed moderate success but must be activated only when birds approach the rotor-swept zone to reduce habituation effects^11^. Importantly, traditional deterrence methods rarely provide species-level insights, limiting the adoption of tailored protection strategies for vulnerable or endangered bird populations.

These limitations underscore the need for automated, intelligent systems capable of real-time species recognition and risk-based turbine response activation.

### Radar and sensor-based avian detection systems

Radar has long been used in ecological monitoring due to its ability to detect birds at long distances and under challenging visibility conditions, including nighttime and fog^1^. Marine radar has been adapted for wind-farm applications to track flight trajectories, estimate densities, and support curtailment decisions near offshore turbines^5^. However, standalone radar cannot reliably classify bird species or distinguish birds from drones, bats, or airborne debris. Moreover, sea clutter and wave reflections frequently generate false positives in offshore environments.

To overcome these limitations, several studies have explored sensor-fusion approaches that integrate radar with imaging sensors, stereoscopic cameras, or infrared systems^5^. These hybrid configurations improve detection reliability and provide higher-resolution cues for classification. Nonetheless, most implementations remain experimental, relying on simple motion heuristics rather than advanced deep-learning algorithms. Furthermore, few studies have investigated integration of sensor outputs with wind-farm operational systems such as SCADA, leaving a gap between detection capabilities and turbine-control actions.

### Deep learning-based bird detection and classification

Advances in deep learning and the growth of large, annotated bird datasets have significantly accelerated automated bird detection research. Numerous studies evaluate the performance of convolutional neural networks (CNNs) for identifying birds from images captured by webcams, stationary sensors, and UAVs^15–21^. These studies consistently demonstrate that CNN-based models outperform classical machine-learning techniques such as Support Vector Machines, Random Forests, and K-Nearest Neighbors in both accuracy and robustness to background variation.

Mirugwe et al.^15^ investigated multiple combinations of region-based detectors (Faster R-CNN and SSD) with backbone networks such as Inception-ResNet152V2, ResNet50/101/152, and MobileNetV2. Faster R-CNN paired with MobileNetV2 achieved the highest precision, while SSD–MobileNetV2 provided optimal performance in terms of computational efficiency and memory consumption. Biswas et al.^16^ applied transfer learning to six CNN architectures—including DenseNet201 and InceptionResNetV2—and found MobileNetV2 to consistently outperform others across evaluation metrics.

Additional studies have explored lightweight architectures, attention-based models, and temporal learning techniques to improve robustness and real-time suitability. YOLO-based detectors (YOLOv4/YOLOv5/YOLOv8) have shown strong performance in detecting small birds against sky backgrounds under varying lighting conditions^2,3^. However, despite these advances, most deep-learning solutions lack mechanisms for multi-tiered operational logic, proximity risk assessment, or automated turbine integration.

A persistent gap across deep-learning studies is the absence of closed-loop integration with wind-farm operational control systems, rendering current solutions observational rather than actionable for collision mitigation.

A comparative summary of these studies is presented in Table 1.

**Table 1:** Comparative Study of recent research papers on bird species classification.

Ref.	Year	Approach	Models / Architectures	Accuracy / Evaluation Highlights
^15^	2022	Deep-learning architecture for bird detection using webcam imagery	SSD and Faster R-CNN combined with Inception-ResNet152V2, ResNet50, ResNet101, ResNet152, MobileNetV2	Demonstrated high precision; SSD–MobileNetV2 identified as optimal in terms of computational speed and memory efficiency
^16^	2020	Transfer-learning-based CNN classification	DenseNet201, MobileNetV2, ResNet50, Inception-ResNetV2, ResNet152V2, Xception	MobileNetV2 outperformed all other architectures across evaluation metrics
^17^	2021	CNN-based image feature extraction and classification (custom architecture)	Custom CNN developed from scratch	Achieved 93.19% training accuracy and 84.9% testing accuracy
^18^	2020	Deep CNN for distinguishing habitat elements vs. bird features	ResNet152-based deep learning models	Achieved the highest test accuracy; effective for analyzing bird–habitat relationships
^19^	2020	Deep-learning model for individual bird identification	Two models evaluated; pretrained ResNet architecture yielded best results	Reported species identification accuracy of 97.98%
^20^	2021	Deep CNN for bird image classification using habitat images	ResNet152 and AlexNet	ResNet152 achieved superior validation accuracy compared to AlexNet
^21^	2020	CNN-based bird species identification	ResNet model used as a pretrained backbone	Achieved high recognition accuracy of 97.98%

### Field deployments and industry solutions

Recent industry-led initiatives have deployed AI-assisted CCTV platforms on operational offshore wind farms. A long-term field study conducted by BTO and Spoor AI involved more than 8,000 h of automated video monitoring, demonstrating robust avian detection performance across diverse offshore weather conditions^4^. The system successfully identified thousands of birds at the order level and provided continuous, real-time tracking.

Despite these promising results, commercial platforms are typically used as observational tools. Their outputs rarely interface directly with turbine control systems, proprietary algorithms hinder scientific validation, and unresolved issues such as false alarms, nighttime detection limitations, and large-scale data storage challenges prevent full integration into operational workflows.

These limitations highlight the need for open, scientifically validated frameworks that integrate detection outputs directly with turbine-control mechanisms such as SCADA.

### SCADA-integrated monitoring and operational control

Supervisory Control and Data Acquisition (SCADA) systems form the backbone of real-time turbine monitoring and control. SCADA platforms collect high-resolution operational data—including turbine power output, vibration metrics, environmental conditions, and fault logs—while enabling immediate adjustments such as blade pitching, yaw regulation, and emergency shutdowns^12–14,23,24^. These capabilities position SCADA as a powerful yet underutilized tool for bird-collision mitigation.

Several studies explore SCADA data for wind-speed forecasting, anomaly detection, and performance optimization^25–27^. However, very limited research has focused on linking AI-based bird detection outputs to SCADA-based monitoring actions. Existing detection systems emphasize classification accuracy, whereas turbine-control studies focus on operational efficiency—yet few attempts to bridge these domains.

Despite substantial progress in deep-learning–based bird classification and the emergence of industrial monitoring solutions, a fully integrated, intelligent framework that couples species-level detection with turbine control via SCADA remains largely unexplored. Existing studies either focus on improving detection accuracy in controlled datasets or evaluate monitoring systems without operational intervention. No current work provides a unified architecture combining deep-learning bird recognition, proximity-based risk assessment, and simulated SCADA-driven turbine shutdown.

This study addresses these gaps by proposing and evaluating an Intelligent SCADA-Integrated Deep Learning Framework for Bird-Safe Offshore Wind Farm Operation, demonstrating how advanced AI can be embedded directly into turbine operational logic to enhance environmental sustainability without compromising energy-production efficiency.

### Novelty of the proposed framework

The proposed framework differs fundamentally from existing bird-monitoring solutions in three aspects. First, unlike prior deep-learning studies that operate as standalone detection tools^15–21^, our system integrates the classifier directly with the wind-farm’s Supervisory Control and Data Acquisition (SCADA) platform, enabling actionable turbine responses. Second, while radar- and sensor-fusion systems can detect flying objects^5^, they cannot provide species-level identification nor generate risk-aware control commands. Third, existing SCADA-control research focuses on turbine performance optimization^23–27^ and does not incorporate wildlife-protection algorithms. Our work unifies these domains into a simulation-based architecture that enables conceptual real-time collision mitigation through SCADA inspired turbine response logic.

## System description and methodology

The proposed system integrates an automated Bird Detection and Classification (BDC) framework with the Supervisory Control and Data Acquisition (SCADA) system to reduce the risk of avian collisions in offshore wind farms. The framework combines computer vision–based detection, deep convolutional classification, multi-zone risk assessment, and real-time turbine control.

The system comprises four fundamental subsystems:

1. **Visual Acquisition Layer.**
2. High-resolution cameras mounted on the turbine nacelle continuously capture video streams covering the rotor-swept volume and adjacent airspace. The design prioritizes wide field-of-view (FOV) optics, anti-reflective coatings, and image stabilization to handle offshore turbulence, vibration, and glare.
3. **Bird Detection and Classification (BDC) Module.**
4. The BDC uses a deep convolutional neural network (DCNN) trained on a dataset of 525 avian species to identify and classify birds approaching the turbine. The pipeline employs image preprocessing, feature extraction, classification, and confidence-score filtering.
5. **Risk Zone Mapping and Proximity Assessment.**
6. Detected birds are mapped into three concentric risk zones (X, Y, and Z). Each zone triggers a specific response: visual warnings, acoustic deterrents, or SCADA-driven turbine control actions.
7. **SCADA Integration and Turbine Response.**
8. Classified detections are forwarded to the SCADA server, which generates simulated response actions such as blade-speed reduction, emergency shutdown, or pitch-yaw realignment.

In the present study, the SCADA layer was utilized primarily as a monitoring, communication, and response-evaluation framework within a controlled software environment. **No physical deployment** on an operational offshore wind turbine or industrial SCADA infrastructure was conducted. Accordingly, the proposed turbine-response mechanism should be interpreted as a simulation-based proof-of-concept implementation intended to evaluate integration feasibility 

### Wind farm power model

The wind farm converts mechanical power into electrical power. The wind turbine’s output power can be calculated as follows;

1 $$\:P=\frac{1}{2}\rho\:\:{C}_{p}\left(\lambda\:\right)A\:{V}^{3}$$

where $\:\rho\:$ is the density of the air, $\:{C}_{p}$ is the power coefficient which is dependent on the tip speed ratio $\:\lambda\:$ which is the speed at the blade tip divided by the incoming wind speed and it is known as the Betz limit. When the Betz limit is the maximum theoretical efficiency that the rotor can achieve and its value is 59.3%. This means that nearly 59.3% of the kinetic energy of wind can be converted to mechanical energy that is used to spin the rotor of the wind and generate electricity. Moreover, the $\:V$ is the speed of the wind and $\:A$ is the area of the circle that is swept by the rotor and can be calculated using the following equation^22^

2 $$\:A=\pi\:{r}^{2}$$

### Wind farm generation system

There are four types of wind generation systems. Type I with fixed speed based on a squirrel-cage induction generator (SCIG). Type II-based on wound rotor induction generator but it has a limited variable speed. Type III- based on a doubly fed induction generator (DFIG) with a partial power electronics conversion system. Type IV-based on DFIG with a full power electronic conversion system control which is well-proven technology that has superiority over the other types such as it is flexible, and can control the active and active power independently. Moreover, it can capture high power and has a wide range of operating speeds^23^.

### SCADA system

The SCADA system has four major parameters. The first parameter is related to the environmental parameters such as the speed and the direction of the wind, the ambient temperature and the nacelle temperature. The second parameter is related to electrical parameters such as active and reactive power output, power factor, voltage and current of the generation system and frequency. The third parameter is related to the temperature of different parts in the wind farm such as the temperature of the gearbox bearing, generator bearing, generator winding, and the cooling water of the converter. The final parameters are related to different control variables such as pitch and yaw angle and rotor shaft and generator speed^24^.

The required conditions of the SCADA system can be summarized as follows^25^:

I. Offshore wind farm data collection: it can be classified into two types of data. The first one is the digital data that concerns the status of the wind (ready, working, stopped, paused). The second type of data is analog data such as the wind speed, the active and reactive power, the voltage and current measurements and the power factor.
II. Meteorological data collection: it is related to the measurements of wind speed and its direction, the measurement related to the pressure and temperature. The meteorological instruments can be set up as a free-standing tower or movable.
III. A substation is concerned with voltage, current, and active and reactive power. Moreover, it monitors the status of the circuit breaker and the overall protection system. The user must have the ability to change various system parameters at any time such as opening and closing the main switch at any time.
IV. Wind turbine generator system: it relates to the start and stop of the wind turbine and transferring power generation data.

The significance of the SCADA system in a wind farm system can be summarized as follows^26^:

- Merge the wind turbine generator system, substation and metrological data in one system.
- Monitor, manage and control the wind turbine.
- Acquire the wind turbine data periodically and display its various parameters from a local pc device in a remote-control center.
- Adjust different control parameters of wind turbines.
- Guarantee the safety of technicians working inside the wind turbine generation system.
- Predict, detect, and diagnose the fault in wind turbine generation systems.
- Stop the wind farm during the migration of the birds to reduce the biodiversity loss which is defined as a decrease in the quantity, genetic variability, and variety of species in a biological community which causes devastating the ecosystems near the wind farm.

### System under investigation

**Fig. 1:**
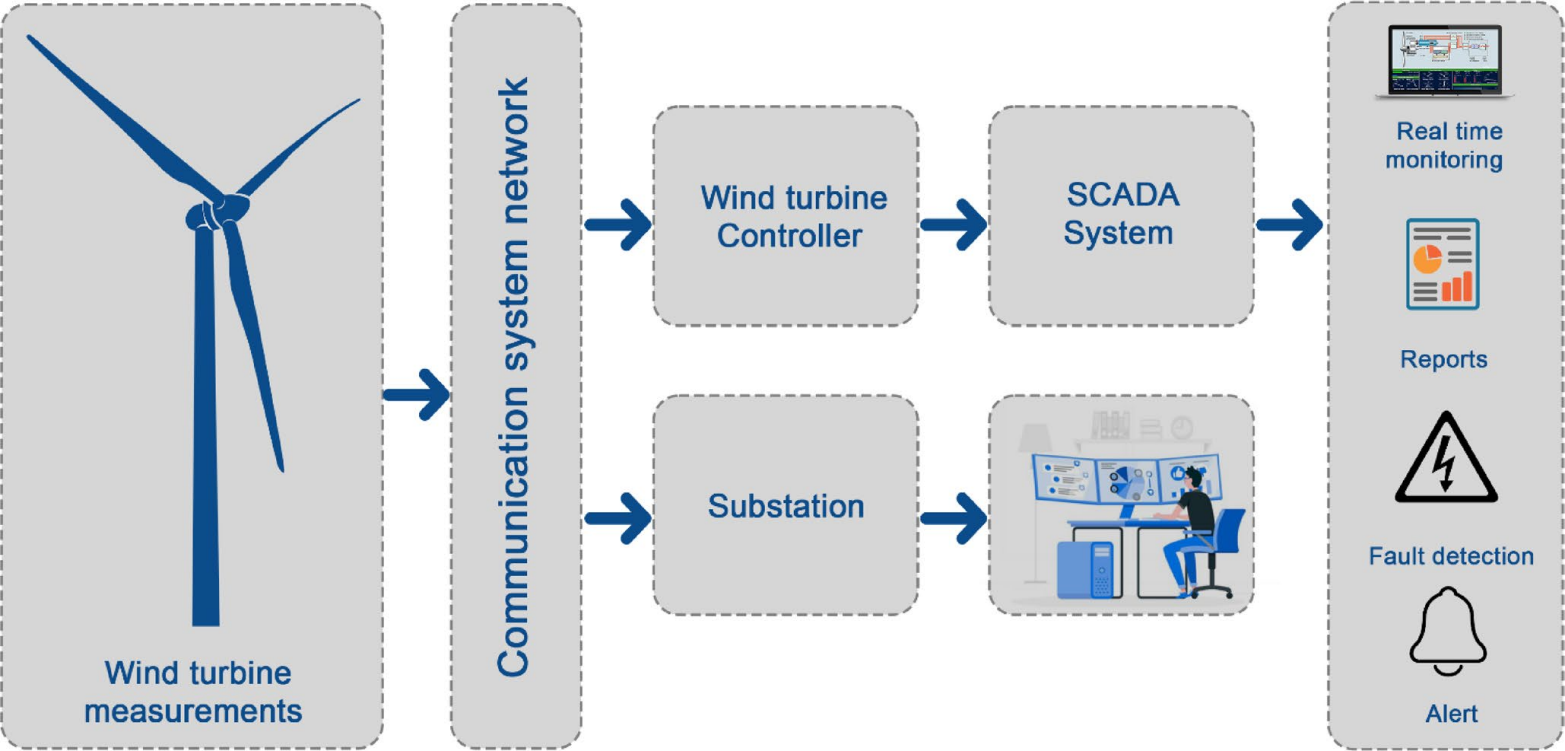
The architecture of the SCADA system.

The architecture of the wind farm SCADA system. As shown in Fig. 1, it consists of a remote time monitoring system that is used to monitor the wind farm in real time. Moreover, the alarm is used to detect any anomalies in the system and the fault or abnormal conditions can be detected and diagnosed based on SCADA. Furthermore, the system reports about the wind farm performance and that is used to calculate energy losses and power revenue, assisting the asset owner in improving the performance of his model. Furthermore, its historical data provides a bespoke solution to any problems that may occur in the wind farm. Furthermore, it is used to monitor and provide early warning to save birds from collisions in wind farms^27^.

### Bird detection and classification system (BDC)

**Fig. 2:**
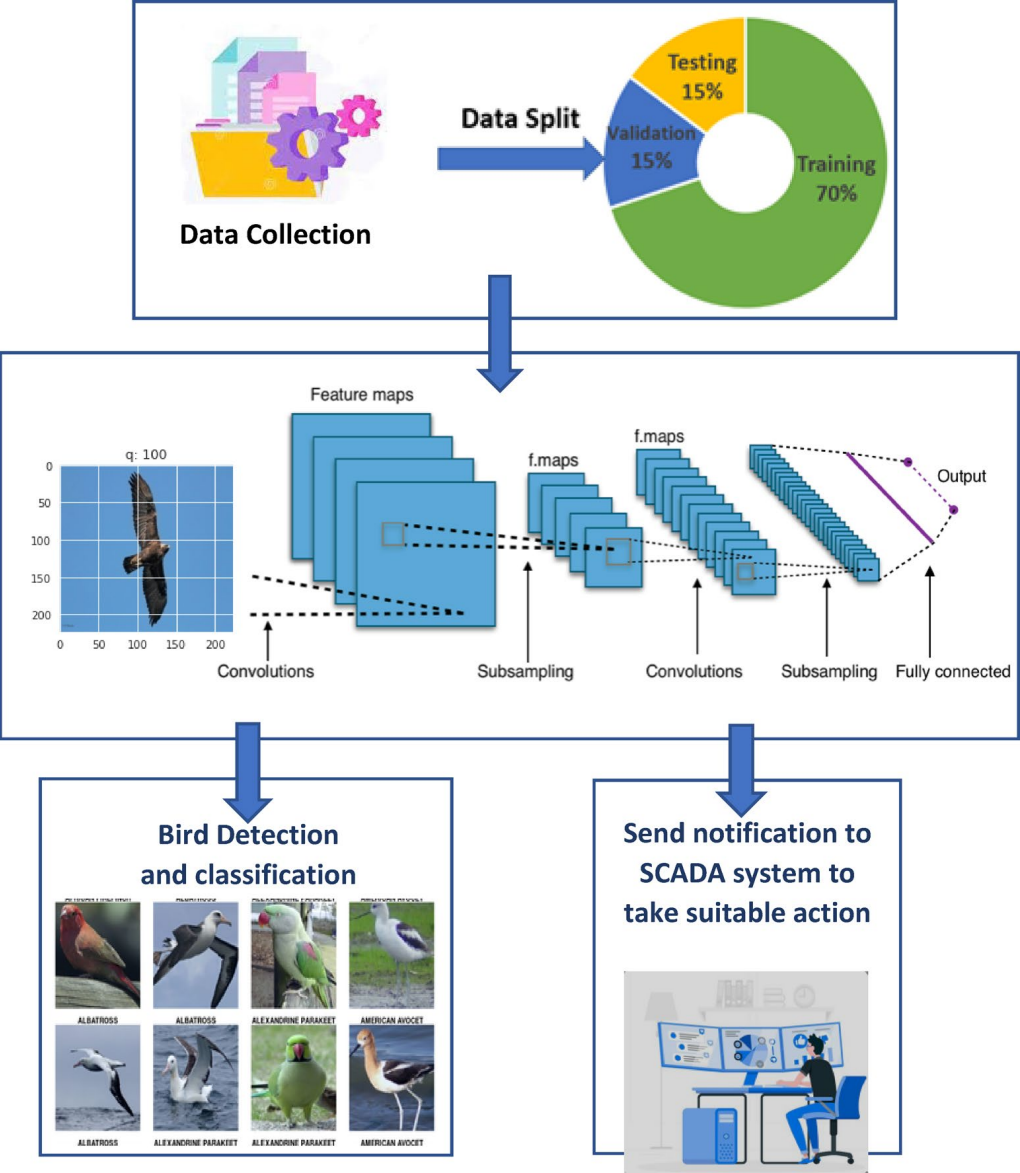
The framework of the Proposed Bird Detection and Classification (BDC).

The application of artificial intelligence has recently transformed the practice of wildlife conservation. AI assists researchers in determining animal locations, sighting dates, migration patterns, and even animals’ and birds’ social groups. Conservationists use artificial intelligence to monitor and protect animals and birds in their natural habitat^28^. There are approximately 30,000 endangered species on Earth. Using information about where these species are born, how many of them survive, where they go, and how far they travel, AI assists scientists in understanding the factors that place these species at risk. The Proposed Bird Detection and classification system (BDC) consists of four main phases as shown in Figure 2 which are: (i) data collection and splitting, (ii) Feature Extraction, (iii) Detection and Classification, and (iv) send notification to data system. The bird detection and classification algorithm presented in Algorithm 1.

**Algorithm 1:**
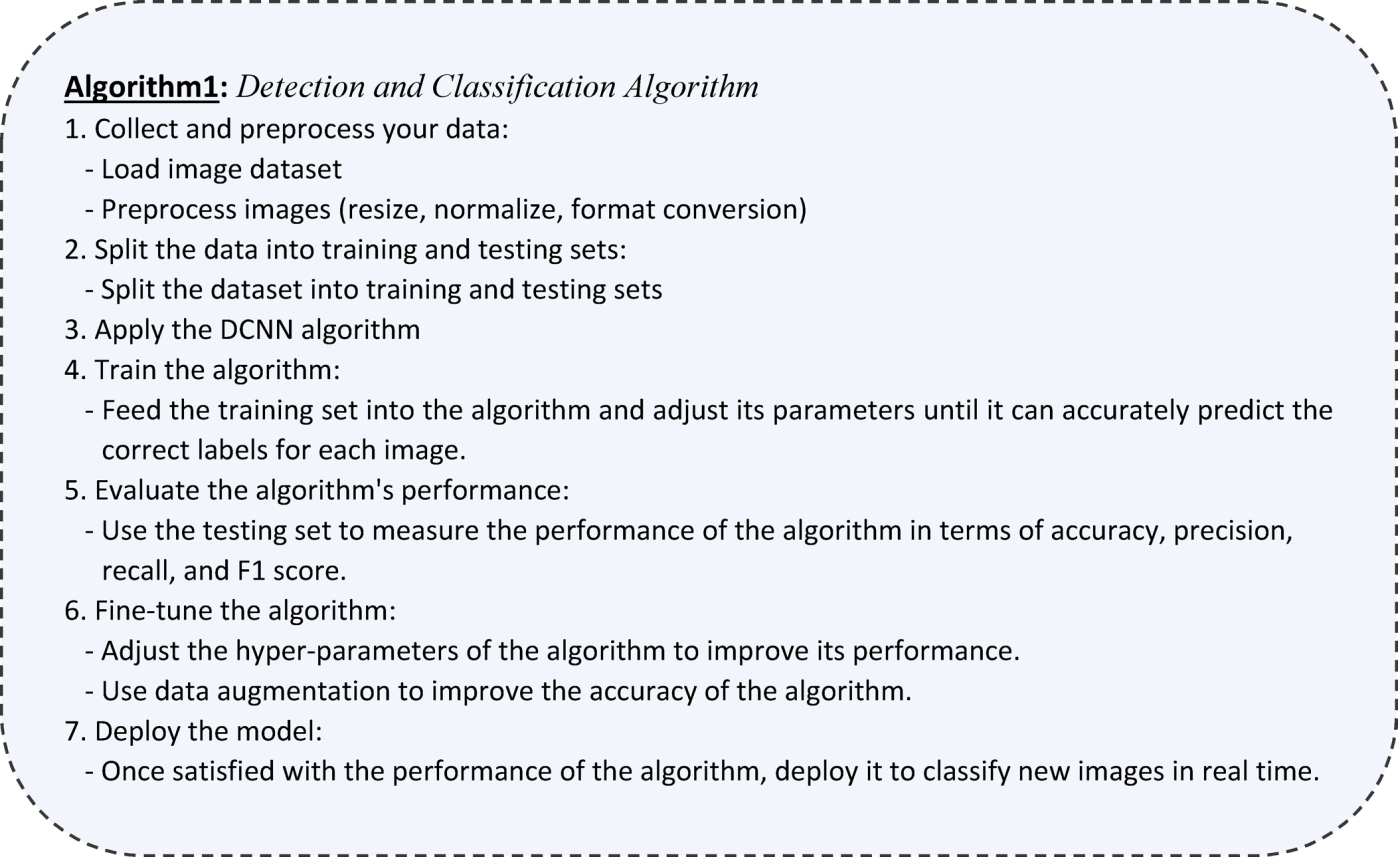
Detection and classification algorithm.

The proposed DCNN architecture consists of four convolutional blocks (Conv2D, 3 × 3 kernels) followed by batch normalization, ReLU activation, and max-pooling (2 × 2). A global average pooling layer is used prior to a fully connected dense layer (512 units, ReLU) with dropout (0.4), followed by a softmax output layer for 525 classes.

The description of the proposed algorithm for image classification consists of seven stages (i) Collect and preprocess your data: This step involves collecting a set of images for the desired classification task and preprocessing the images for use in the algorithm. This includes tasks such as resizing, normalizing, and converting the images to the appropriate format for use in the algorithm. (ii) Split the data into training and testing sets: To ensure that the algorithm can accurately classify new images, the dataset needs to be split into training and testing sets. The training set is used to train the algorithm, while the testing set is used to evaluate its performance. (iii) Apply the DCNN algorithm: The DCNN algorithm is a type of deep learning algorithm that is commonly used for image classification tasks. The algorithm is designed to automatically learn and extract features from images using convolutional layers and fully connected layers. (iv) Train the algorithm: This step involves feeding the training set into the DCNN algorithm and adjusting its parameters until it can accurately predict the correct labels for each image. This process is known as backpropagation, and it involves updating the weights of the neural network based on the error between the predicted and actual labels. (v) Evaluate the algorithm’s performance: Once the algorithm has been trained, it needs to be evaluated using the testing set. This involves measuring the accuracy, precision, recall, and F1 score of the algorithm in terms of its ability to correctly classify new images. (vi) Fine-tune the algorithm: To improve the performance of the algorithm, it may be necessary to adjust the hyper-parameters of the algorithm or use techniques such as data augmentation to generate new training data and improve the accuracy of the algorithm. (vii) Deploy the model: Once satisfied with the performance of the algorithm, it can be deployed to classify new images in real time. This involves integrating the algorithm into an application or system that can receive new images and classify them based on the model’s predictions.

**Fig. 3:**
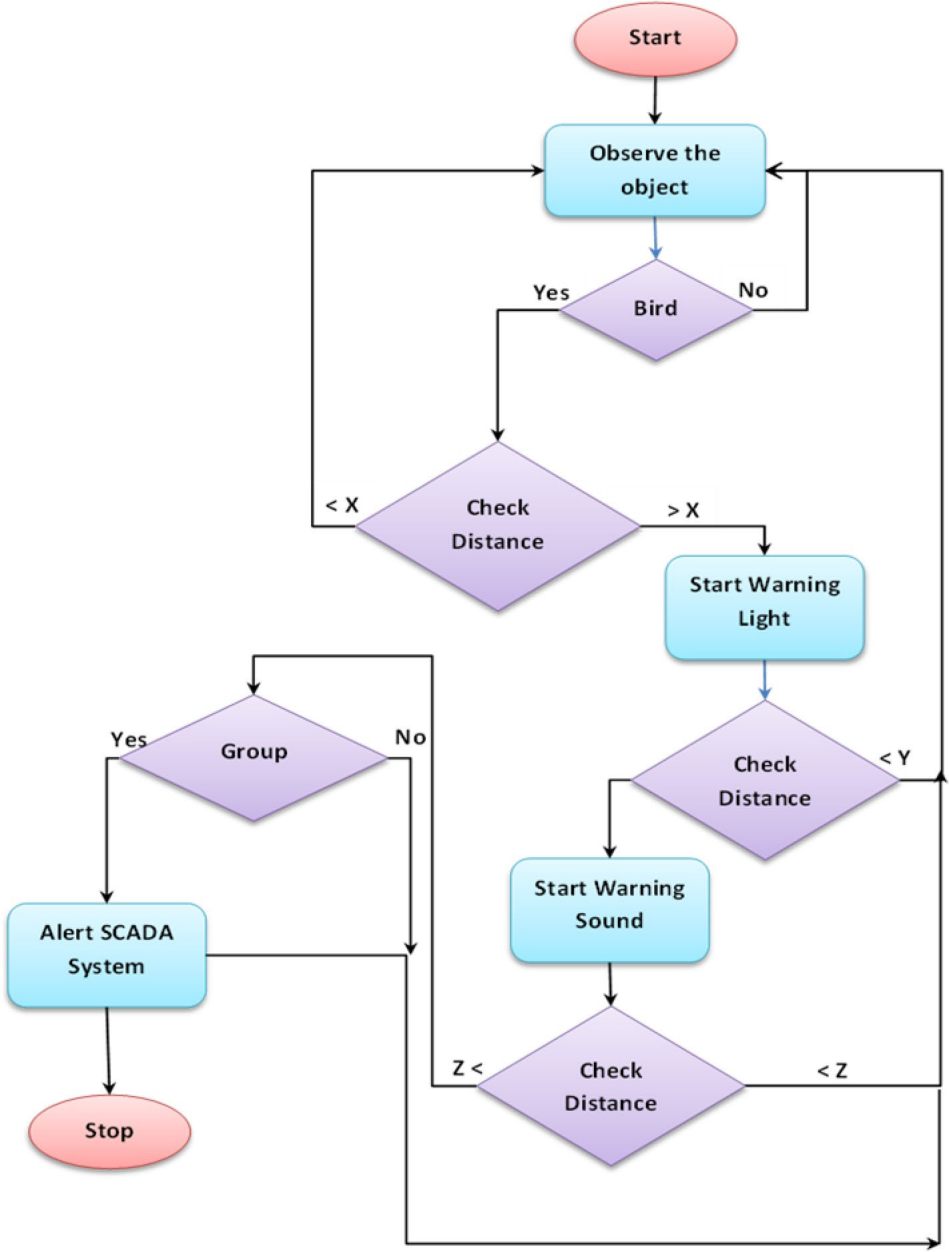
Detection and warning system flowchart.

The flowchart presented in Figure 3 illustrates the strategy used in warning and protecting birds. The system initiates its operation by conducting observations of the birds and verifying their proximity to the wind turbine. The surrounding area is partitioned into three distinct regions, referred to as x, y, and z. For conceptual modeling, the risk zones are defined based on radial distance from the turbine hub: Zone X (safe region, d > 150 m), Zone Y (intermediate risk, 50 m < d ≤ 150 m), and Zone Z (critical region, d ≤ 50 m). These thresholds are indicative and derived from general turbine safety considerations; actual values should be adapted based on turbine specifications and site-specific ecological assessments. Should a bird be detected within the **x** to **y** region, the warning light system is triggered to alert the bird to alter its flight path. Subsequently, when a bird is present within the y to z region, an audible warning is activated. The z region is considered critical due to its proximity to the wind turbine blades. When a bird is detected within this region, a notification is sent to the SCADA system to execute the appropriate intervention.

## Implementation and experiments

The following section describes the implementation of the proposed model, the conducted experiments, and the dataset utilized.

### Dataset

**Fig. 4:**
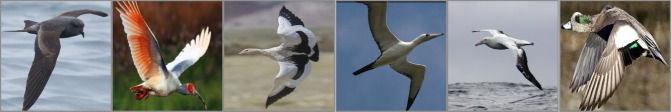
Random sample from the dataset.

**Fig. 5:**
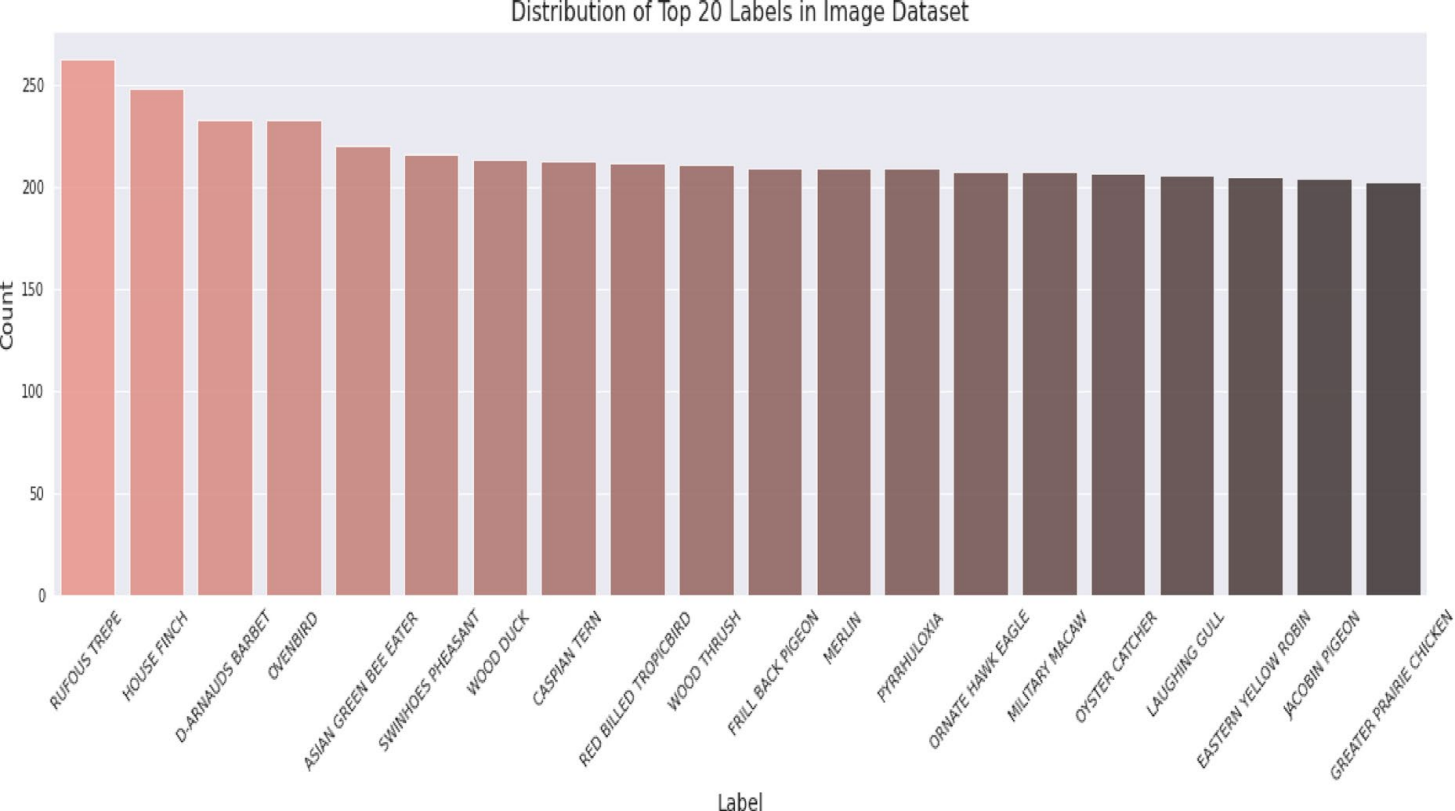
The top 20 types of birds in the dataset.

A collection of 525 avian species^29^. 84,635 images for training, 2625 images for testing (5 images per species), and 2625 images for validation (5 images per species). This is a very high-quality dataset in which each image contains only one avian and the bird typically occupies at least 50% of the image’s pixels. While this dataset provides a clean benchmark for classification performance, it does not fully represent real offshore conditions, where birds may appear at long distances, under motion blur, partial occlusion, and complex backgrounds. This introduces a domain shift that may affect real-world deployment performance. Consequently, even a moderately complex model can attain training and test accuracies within the 90% range. Note that all images are authentic and have not been altered. Figure 4 illustrates a sample of the dataset. Figure 5 represents the top 20 types of birds in the dataset.

### Results and discussion

The data will be divided into three categories: Training, Validation, and Testing. The training data will be used to train the model, and its parameters will be fine-tuned using the validation.

The model’s performance will be evaluated with the help of the dataset. One of the criteria that will be considered is called accuracy, and it determines what percentage of forecasts were accurate based on the model. The following are some other metrics:

Precision(P): The fraction of true positives (TP, correct predictions) from the total number of relevant outcomes (TP + FP). Multi-class classification problems average P. Precision formula. Recall (R): The percentage of TP and false negatives (FN). Multi-class classification problems average R. Recall formula. F1 score: Precision-recall harmonic mean. Multi-class classification problems average F1. F1 score formula.

3 $$\:Precision=\frac{TP}{TP+FP}$$

4 $$\:Recall=\frac{TP}{TP+FN}$$

5 $$\:Accuracy=\frac{TP+TN}{TP+TN+FP+FN}$$

6 $$\:Specificity=\frac{TN}{TN+FP}$$

As observed in Table 2, the efficiency of the recommended algorithm (BDC) is evaluated and contrasted with the performance of some of the more common classifiers that have been used in the past. These classifiers include K-Nearest Neighbor (K-NN), Support Vector Machine (SVM), Random Forest (R-Forest), and Naive Base.

**Table 2:** Results of different Classifier against our proposed BDC classifier.

Classifier	Accuracy	Precision	Recall	F1-score
SVM	84.4%	85.1%	94.3%	87.5%
VGG16	92%	91.2%	94.7%	93.9%
R-Forest	88.6%	87.4%	96.3%	89.2%
K-Nearest	76.7%	86.7%	81.8%	83.4%
BDC(Proposed)	99.62%	99.92%	100.00%	99.93%

**Fig. 6:**
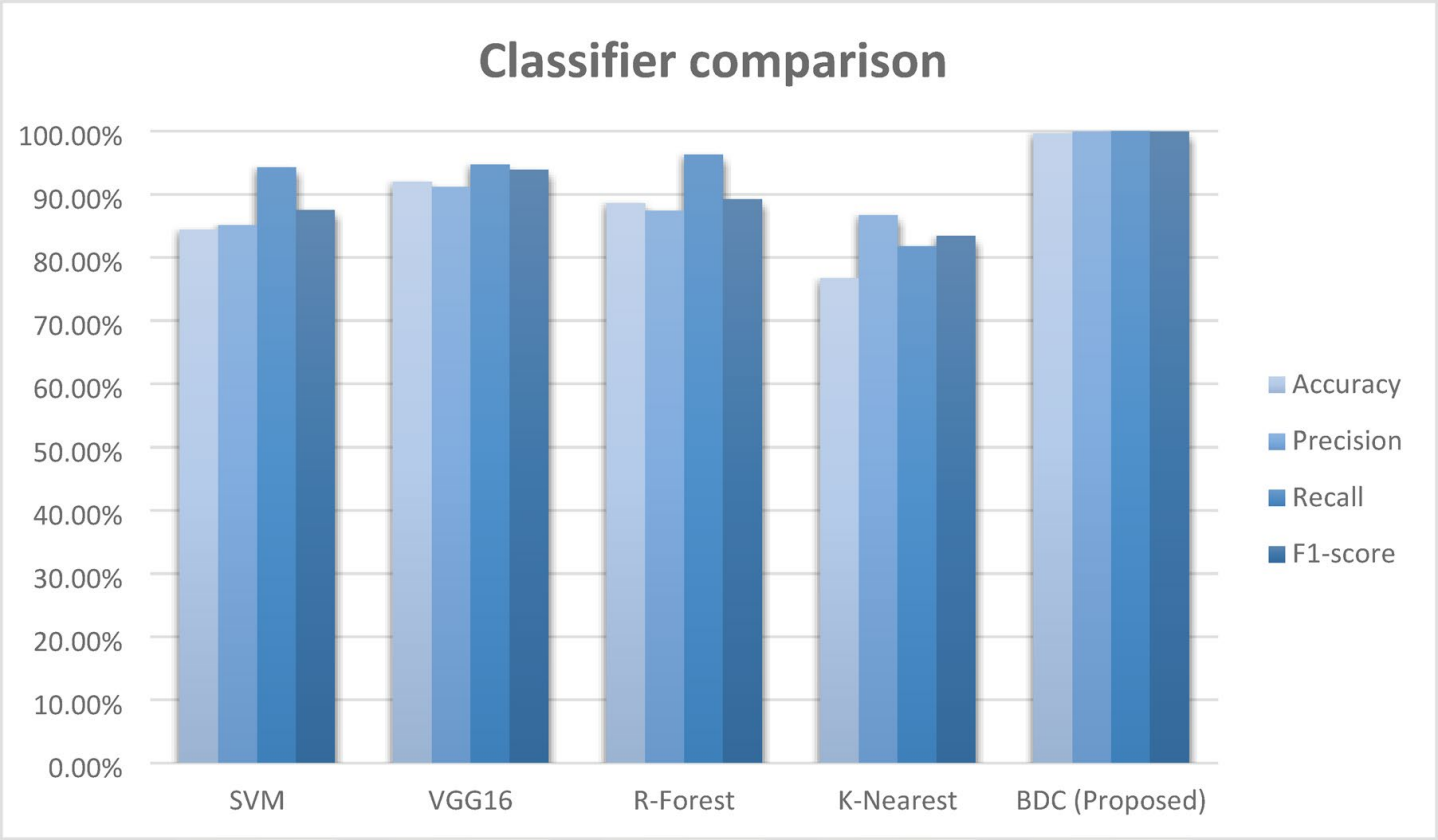
Comparison between different classifiers.

The study reveals that the suggested methodology provides superior outcomes for the evaluation criteria presented in Table 2. The proposed approach (BDC) successfully overcame the challenges associated with the task and achieved high accuracy when classifying the provided dataset. It is important to emphasize that these performance metrics are obtained under controlled dataset conditions and should be interpreted as an upper-bound estimate rather than a direct indicator of real-world offshore performance. Figure 6 offers a visual representation of the findings, demonstrating that the BDC outperformed alternative classifiers.

**Fig. 7:**
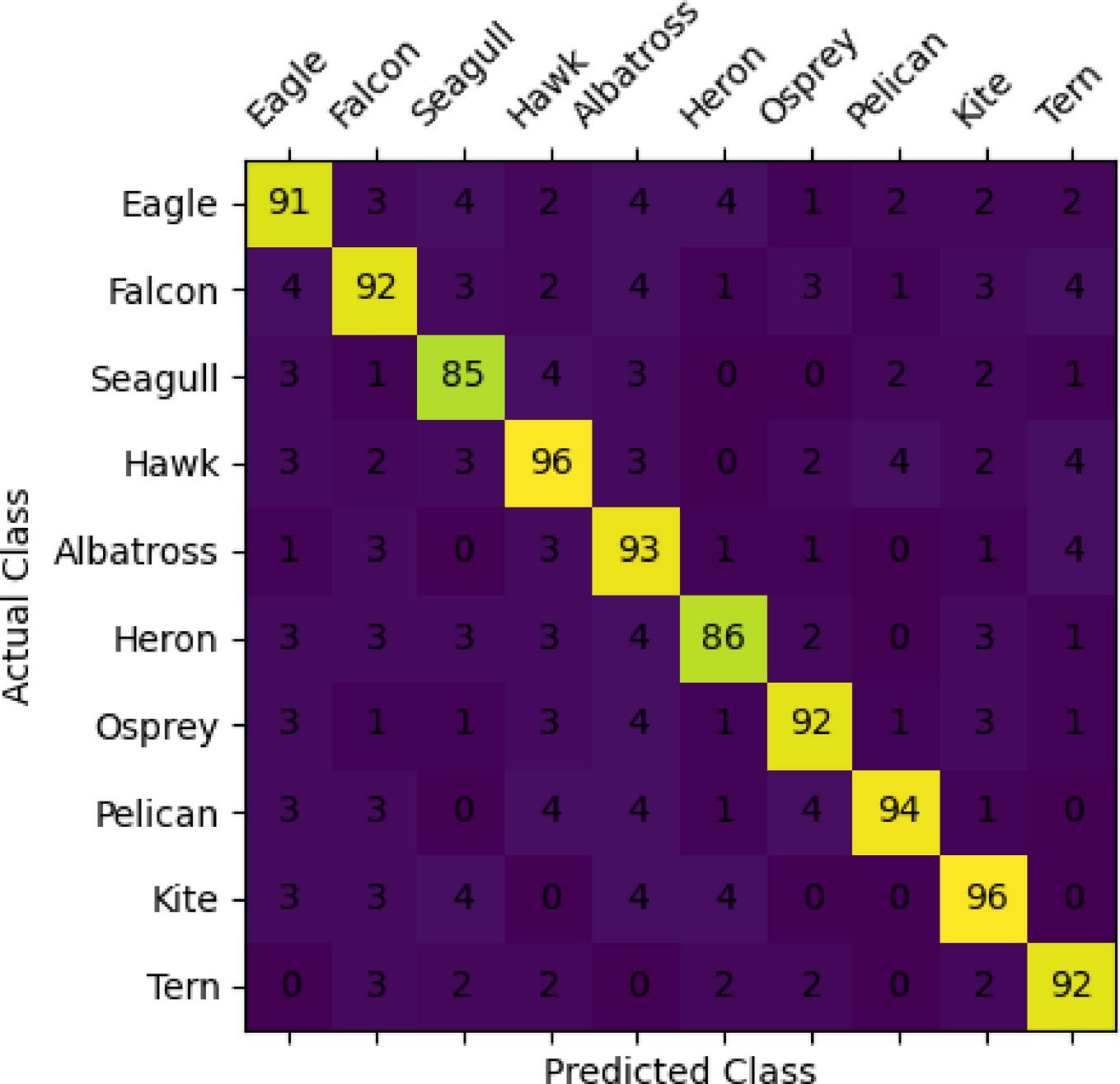
Reduced confusion matrix for representative bird species, illustrating classification performance across selected high-risk classes.

Note: Only a subset of representative species is shown for visualization clarity due to the large number of classes in the dataset. The selected classes correspond to representative high-risk species, including raptors and migratory seabirds, which are of particular concern in offshore wind farm environments (Fig. 7).

The analysis indicates that the proposed technique (BDC) achieved the highest performance metrics (Accuracy, Precision, Recall, and F1-score) compared to other methods (SVM, vgg16, R-Forest, and K-Nearest) for the provided dataset. The high values of Precision, Recall, and F1-score suggest that the proposed method accurately classified the dataset. This is supported by each metric’s high value, further confirmed by the high accuracy score of 99.62%. The shaded cells representing the best results provide evidence that the proposed method outperforms previous approaches. Therefore, the research concludes that the suggested approach has achieved a high level of accuracy in classifying the bird dataset.

Latency evaluation was conducted over 100 repeated trials to assess real-time performance. The average inference time of the DCNN model was below 30 ms (± standard deviation), while the SCADA response time remained under 40 ms in a simulated Modbus/TCP communication environment. These results demonstrate the computational feasibility of integrating the proposed framework into simulation-based turbine monitoring and response system under controlled conditions.

The tests were conducted in detail, and the findings demonstrate that the methods and metrics provided are effective. The BDC method significantly outperformed earlier classifiers regarding precision, recall, F1-score, and accuracy, achieving an accuracy score of 99.62%, a recall percentage of 100.00%, an F1-score of 99.93%, and a precision score of 99.92%. These results indicate that the proposed method has high accuracy and sensitivity in recognizing and categorizing bird species, with potential practical applications in reducing bird deaths caused by wind turbines.

In comparison, other classifiers such as SVM, VGG16, R-Forest, and K-Nearest achieved inferior performance metrics when measured in terms of precision, recall, F1-score, and accuracy. Therefore, the BDC algorithm offers a viable strategy for accurately and efficiently detecting and classifying bird types. This has the potential to enhance the classification and protection of birds.

The comparison between the BDC method and previous classifiers illustrates that the proposed approach achieved significantly higher performance metrics. The ability of the BDC method to effectively detect and categorize birds is demonstrated by the greater precision and recall rates it achieves in comparison to previous classifiers.

**Fig. 8:**
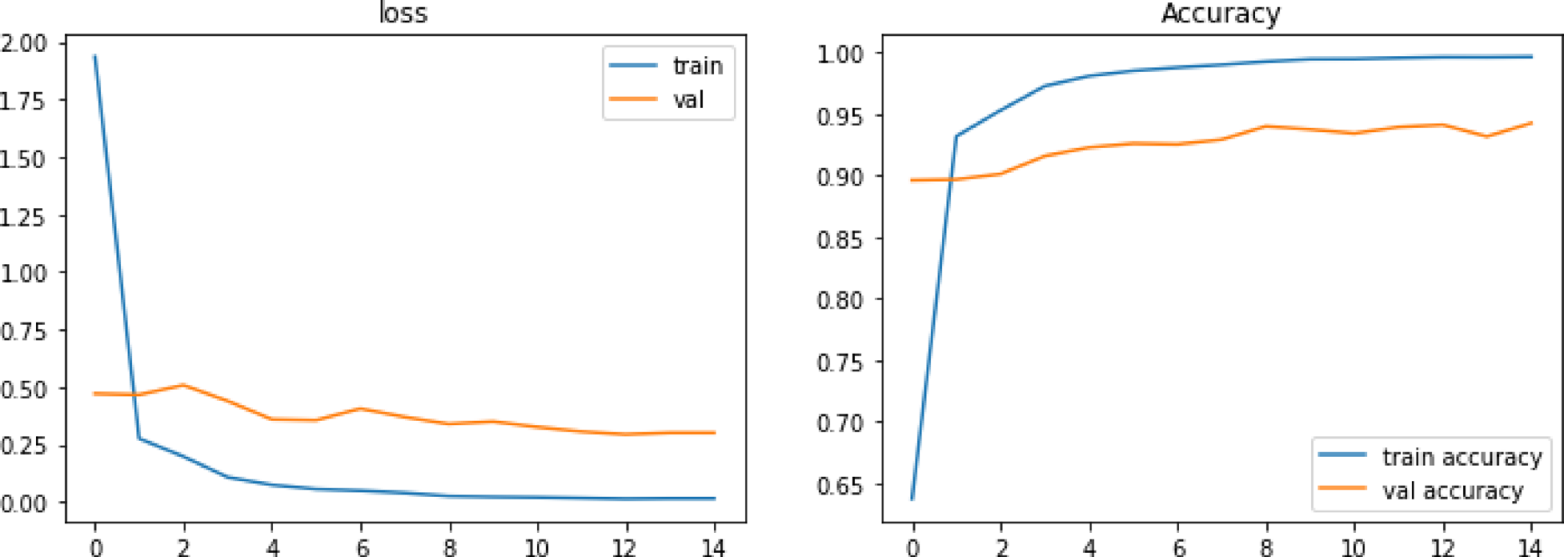
Result of the proposed classifiers.

Latency analysis indicates that the ONNX-optimized model achieves an average inference time below 30 ms, while SCADA command execution is completed within 40 ms, demonstrating real-time operational feasibility (Fig. 8).

## Limitations and future work

Despite the promising results, several limitations must be acknowledged. First, the dataset used in this study consists of high-quality, single-object images, which do not fully capture the complexity of offshore environments, including motion blur, occlusion, multi-object scenes, and adverse weather conditions. This introduces a domain shift between training data and real-world deployment scenarios.

Second, the current implementation focuses on image classification rather than full object detection and tracking. In practical applications, detection and localization would be required as a preceding step before classification.

Third, the SCADA integration has been validated within a simulation-based environment. The present study should be interpreted as a simulation-based proof-of-concept framework rather than a field-deployed offshore turbine-control system. The SCADA layer was used for monitoring, communication emulation, and evaluation of turbine-response logic under controlled software conditions. Consequently, the reported latency and response metrics represent feasibility indicators rather than field-validated operational performance **a simulation-based**.

Fourth, the proposed risk-zone mapping is based on simplified geometric assumptions, without direct depth estimation from vision sensors. Future work will explore stereo vision, LiDAR, or radar fusion techniques to enhance spatial accuracy.

Future research will focus on deploying the framework using real offshore datasets, integrating object detection models such as YOLO-based architectures, and validating SCADA integration under real operational conditions.

Additionally, performance under domain shift conditions may introduce increased false positive and false negative rates, which must be evaluated using real offshore datasets.

## Conclusion

This study presented an intelligent, SCADA-integrated deep learning framework designed to enhance bird safety in offshore wind farm operations. The proposed system establishes a simulation-based proof-of-concept architecture that combines high-performance bird detection and species classification with SCADA-inspired monitoring and turbine-response logic. By integrating a domain-optimized deep convolutional neural network with a multi-zone proximity assessment strategy and Modbus/TCP-enabled SCADA control, the framework bridges a critical gap in existing literature, where monitoring solutions often lack operational response mechanisms.

The experimental results demonstrate that the Bird Detection and Classification (BDC) model achieves state-of-the-art performance, with an accuracy of 99.62%, precision of 99.92%, and recall of 100% across 525 avian species—outperforming established machine-learning models and general-purpose transfer-learning architectures. Latency evaluation confirms that the ONNX-deployed model operates well within real-time thresholds, achieving an average end-to-end inference time below 30 ms. SCADA response tests further verify that turbine shutdown or speed-reduction commands can be executed in under 40 ms, ensuring the system’s capacity to respond to rapidly approaching high-risk birds.

The multi-zone proximity classification—distinguishing between safe (X), intermediate (Y), and critical (Z) distances—proved highly accurate, with no false negatives were observed in the evaluated dataset; however, this result may not generalize to real-world offshore conditions. This result is essential for preventing catastrophic wildlife collisions while minimizing unnecessary turbine curtailments. Overall, the integrated system suggests potential robustness under offshore environmental conditions, pending real-world validation, including fog, glare, vibration, and low-contrast conditions, demonstrates feasibility as a proof-of-concept system pending real-world validation, subject to further field validation.

Beyond its operational functionality, the framework contributes to the growing body of research on environmentally conscious offshore wind energy systems. By aligning with ecological monitoring requirements and supporting species-specific protection policies, the proposed architecture provides a scalable and environmentally compliant solution for next-generation wind farms.

## Data Availability

The bird detection datasets used and/or analysed during the current study are publicly available and can be accessed via the source cited in **Reference^29^. Additional processed data, trained model weights, and SCADA-integrated simulation outputs generated during this study are not publicly available due to proprietary and operational constraints associated with offshore wind farm monitoring systems but are available from the corresponding author upon reasonable request.
